# Public Opinion Perceptions, Private Support, and Public Actions of US Adults Regarding Gun Safety Policy

**DOI:** 10.1001/jamanetworkopen.2020.29571

**Published:** 2020-12-22

**Authors:** Graham Dixon, Kelly Garrett, Mark Susmann, Brad J. Bushman

**Affiliations:** 1 School of Communication, The Ohio State University College of Arts and Sciences, Columbus; 2 Department of Psychology, The Ohio State University College of Arts and Sciences, Columbus

## Abstract

**Importance:**

Despite broad public support for gun safety policies, minimal policy implementation has occurred.

**Objective:**

To investigate factors that encourage greater private support for and public action on gun safety policy.

**Design, Setting, and Participants:**

Three studies were conducted: a public opinion survey (Study 1) was conducted from January 8 to 22, 2019, and 2 experiments (Studies 2 and 3) were conducted from August 27 to October 17, 2019, and April 15 to 21, 2020, respectively. Adults living in the US were eligible to participate in Studies 1 and 3. Students 18 years and older participating in a research experience program were eligible to participate in Study 2. Study 1 was administered online by Ipsos, a market research company. A nationally representative sample of 1000 US adults was obtained from Ipsos’ online KnowledgePanel, of whom 508 completed the public opinion survey. For Study 2, which was conducted in a university laboratory, 354 participants were recruited from a university research pool, all of whom completed the study. Study 3 was administered online by the market research company YouGov, which identified 727 US gun owners from its opt-in panel, from which it constructed a census-matched sample of 400 participants.

**Exposures:**

Participants read a statement about the 2018 Marjory Stoneman Douglas High School shooting. Content was manipulated as a 2 (corrective information vs no corrective information) × 2 (system changeable vs system not changeable) between-subjects factorial design with random assignment. The corrective information included polling data highlighting widespread support among gun owners for several gun safety policies. System changeable described gun safety policies passed by Florida’s legislature.

**Main Outcomes and Measures:**

Main outcomes were support for gun safety policies and public disclosure of support.

**Results:**

The 3 studies included a total of 1262 participants (Study 1: 508 participants; weighted mean [SD] age, 47.7 [17.5] years; 261.9 women [51.6%]; 82.5 Hispanic [16.2%] and 60.3 Black [11.9%]; Study 2: 354 participants; mean [SD] age, 20.0 [2.3] years; 232 women [65.9%]; 100 Asian [28.3%] and 37 Black [10.5%]; Study 3: 400 participants; weighted mean [SD] age, 52.1 [16.4] years; 187.3 women [46.8%]; 295.5 White [73.9%], 44.5 Hispanic [11.1%], and 32.4 Black [8.1%]). Study 1 found that 63% to 91% of gun owners and 83% to 93% of non–gun owners supported key gun safety policies, yet both groups significantly underestimated gun owners’ support for these policies by between 12% and 31%. Studies 2 and 3 found that exposure to corrective information was associated with a small increase in support for 2 gun safety policies of between 4% and 15%, both in terms of participants’ privately held beliefs and the beliefs they would be willing to share publicly.

**Conclusions and Relevance:**

This survey study found that many US adults failed to recognize that most gun owners support key gun safety policies. Correcting this misperception was associated with greater private and public support for gun safety policy.

## Key Points

**Question:**

Is correcting misperceptions on gun safety public opinion associated with an increase in private support for and public actions on gun safety in the US?

**Findings:**

In this 3-part survey study with a total of 1262 respondents, a nationally representative survey found that a majority of gun owners and non–gun owners support key gun safety policies, yet both groups significantly underestimate gun owners’ support for these policies. The 2 additional survey experiments, including one of university students and one with a representative sample of gun owners, found that corrective information about gun owners’ support for gun safety policies was associated with a significant increase in participants’ support for the policies both privately and publicly.

**Meaning:**

These findings suggest that increasing awareness of the high levels of support for gun safety policies among gun owners may lead individuals to be more supportive of the policies both in private and in public.

## Teaser

This 3-part survey study assesses whether correcting the public’s misperceptions of gun safety is associated with greater private support for and public actions on gun safety.

## Introduction

Gun violence is a public health crisis in the US, with over 255 000 deaths recorded between 2012 and 2018.^1^ Policies aimed at curbing gun violence are widely supported by the American public. Polls show that an overwhelming majority of US adults—gun owners and non–gun owners alike—support expanded background checks, mandatory waiting periods, and requirements for safe storage of firearms.^2,3,4,5^ Despite this support, many gun owners express apprehension about enacting new gun safety laws, and they tend to favor political candidates who reject such policies.^5,6^ Furthermore, supporters of gun safety measures show less willingness than gun rights advocates to express their views, sign petitions, and contact elected officials.^7^

Misperceptions of the gun safety opinion climate may help to explain the disconnect between policy support and policy action. Individuals who perceive themselves to be in the minority are less likely to express their views publicly, regardless of whether their perception is accurate.^8^ Although individuals often overestimate public support for their positions, that is unlikely in this case.^9^ In the US, news media regularly misrepresent public views on gun policy, inaccurately suggesting a deep divide between gun owners and non–gun owners.^2^ This misrepresentation could give rise to pluralistic ignorance, whereby individuals whose attitudes and judgments are shared by a majority erroneously believe that they are in the minority,^10,11^ which could lead people to overestimate polarization about gun safety policy more generally.^12^

Pluralistic ignorance and false polarization can have a profound effect on how people evaluate and voice their private views. Pluralistic ignorance can lead people to adjust their attitudes and behaviors toward a misperceived norm and to reduce their willingness to disclose their private views on the issue in public.^10,11,13,14,15,16,17^ Social conformity also plays an important role in this process, because individuals often display an aversion to expressing beliefs that contradict a perceived norm among in-group members.^18,19^ Individuals also self-censor to avoid anticipated negative social consequences associated with expressing a viewpoint that deviates from the in-group’s position, such as a loss of respect or lower perceived competence.^11,20^ Such self-censorship sometimes takes the form of political nonparticipation.^20^

Communicating accurate information about gun owners’ views toward gun policies could help correct opinion-climate misperceptions, which could make it more likely that US adults—especially gun owners—will publicly express their support for gun safety policies.^11,15^ For gun owners, emphasizing congruence between their private views and the views of others could reduce the threat of pluralistic ignorance resulting from in-group misperceptions. For non–gun owners, it could reduce the risk of false polarization resulting from outgroup misperceptions.

Conveying opinion-climate information is not without risk. If individuals perceive a message to be a persuasive appeal or a form of propaganda, then boomerang effects are more likely.^21^ Individuals respond negatively to being told what to think or how to behave, and this can cause their beliefs and attitudes to become more entrenched. In this case, however, the risk appears modest. Learning that gun owners generally support gun safety policies will be congruent with most recipients’ private viewpoints, whether they own guns or not. Motivations to push back against such messages should be limited.

The benefits of correcting misperceptions about the gun policy opinion climate may be contingent on other factors. Those who take a position endorsed by the in-group often avoid challenging the status quo if they think change is unlikely or impossible.^22,23,24^ Conversely, individuals are more likely to challenge the status quo when they perceive that it can be or has been changed.^25^ In the context of this study, we argue that correcting misperceptions of public opinion about gun safety will be uniquely effective among individuals primed to think that change is possible.

A history of federal inaction on gun safety in the wake of deadly mass shootings has left many US adults with the impression that changing gun laws will be slow and difficult, perhaps even impossible.^26^ Still, there is some evidence that change is possible. For example, Florida’s state legislature passed a law broadening firearm restrictions and implementing a mandatory waiting period following the Marjory Stoneman Douglas High School shooting in 2018.^27^ Calling attention to this fact might augment the effect of providing accurate information about public opinion toward gun safety policies.

To test these predictions, we conducted a series of 3 studies that documents the presence of misperceived opinion climates and examines whether correcting these misperceptions, with or without emphasizing system change, influences attitudes and actions on gun safety policy.

## Study 1

### Methods

The marketing research company Ipsos conducted a 15-minute (median length) omnibus survey with a nationally representative sample of US adults from January 8 to 22, 2019. Questions for the current project were presented in the last of 3 survey blocks, each of which concerned a different topic. No participants skipped the whole block, and 2% or less skipped questions used in these analyses. There is no evidence that participants’ demographic characteristics influenced these missing data. Ipsos sampled households from its KnowledgePanel, a probability-based web panel designed to be representative of the US (eAppendix 1 in the Supplement). Of the 1000 participants invited to participate, 508 (50.8%) completed the main survey (the percentage recommended for probability-based internet panels). The company provided poststratification weights that it computed using an iterative proportional fitting (raking) procedure using data from the March 2018 supplement of the Census Bureau’s Current Population Survey. We used these survey weights in all computations. This and all the studies in this survey followed the American Association for Public Opinion Research (AAPOR) guidelines. The study had the approval of the Ohio State University institutional review board. All participants provided consent via an electronic form before completing the survey.

We assessed participants’ knowledge and attitudes about 3 gun safety policies: universal background checks, mandatory waiting periods, and safe storage laws. We described each topic with wording used in prior studies: “Requiring a background check for all gun sales to make sure a purchaser is not legally prohibited from having a gun”^2^; “Imposing a federal mandatory waiting period on all gun purchases, so that everyone who purchases a gun must wait a certain number of days before taking the gun home”^4^; and “Requiring by law that a person lock up the guns in their home when not in use to prevent handling by children or teenagers without adult supervision.”^2^ We asked 2 kinds of questions about each topic. First, we asked, “To what extent do you support or oppose the following policies?” (1 = strongly opposed to 6 = strongly supportive), which we dichotomized (support or oppose) in order to estimate the proportion of the population supporting each policy. Given the high-profile topic, the absence of a neutral midpoint for these items reduces the risk of participant satisficing, and has been shown to improve validity and reliability.^28^ Second, we asked, “What percent of American gun owners do you think support the following policies?” Participants entered their estimate into a textbox that accepted values from 0 to 100. All analyses were conducted from May 25 to October 16, 2020, using Stata/IC, version 14.2 (StataCorp LLC). All *P* values were 2 sided, and a *P* value of less than .05 was considered significant.

### Results

The 508 participants in the public opinion survey included 261.9 women (51.6%) and was racially diverse (82.5 [16.2%] Hispanic and 60.3 [11.9%] Black participants), and the age distribution was comparable with the national average (mean [SD] = 47.7 [17.5] years). More generally, weighted participant demographic details were comparable with the US population (Table). Importantly, gun owners represented 30.2% of the sample (n = 150.2), mirroring Gallup’s recent finding in a nationally representative poll.^29^

**Table.** Sample Demographic Details for All Studies

Characteristic	No. (%)		
	Study 1: national sample^a^ (n = 508)	Study 2: student sample^b^^,^^c^ (n = 354)	Study 3: gun owners^a^ (n = 400)
Age, y			
18-29	107.1 (21.1)	350 (98.9)	37.4 (9.4)
30-44	127.1 (25.0)	4 (1.1)	115.4 (28.9)
45-59	128.8 (25.4)	0 (0)	102.0 (25.5)
≥60	145.0 (28.6)	0 (0)	145.2 (36.3)
Female	261.9 (51.6)	232 (65.9)	187.3 (46.8)
Race/ethnicity			
White, non-Hispanic	322.5 (63.5)	206 (58.2)	295.5 (73.9)
Black, non-Hispanic	60.3 (11.9)	37 (10.5)	32.4 (8.1)
Other, non-Hispanic	35.9 (7.1)	111 (31.4)	23.9 (6.0)
Hispanic	82.5 (16.2)	NA	44.5 (11.1)
≥2 Races, non-Hispanic	6.7 (1.3)	NA	3.6 (0.9)
Education			
Less than high school	55.4 (10.9)	0	13.4 (3.4)
High school	145.5 (28.6)	0	136.7 (34.2)
Some college	143.2 (28.2)	354 (100)	138.8 (34.7)
Bachelor’s degree or higher	163.9 (32.3)	0	111.2 (27.8)
Geographic location			
Northeast	90.2 (17.8)	NA	42.4 (10.6)
Midwest	105.6 (20.8)		88.8 (22.2)
South	191.5 (37.7)		203.7 (50.9)
West	120.6 (23.8)		65.1 (16.3)
Political party affiliation			
Democrat	147.8 (30.7)	NA	84.3 (21.1)
Independent	116.4 (24.2)		127.0 (31.8)
Republican	116.6 (24.3)		161.5 (40.4)
Other	100.2 (20.8)		27.1 (6.8)
Gun ownership			
Owners	150.2 (30.2)	13 (3.7)	400 (100)

Abbreviation: NA, not applicable.

^a^ Proportions computed using survey weights.

^b^ Questionnaire did not include geographic location or party affiliation measures.

^c^ Race question did not include multiracial or Hispanic options.

**Figure 1.**
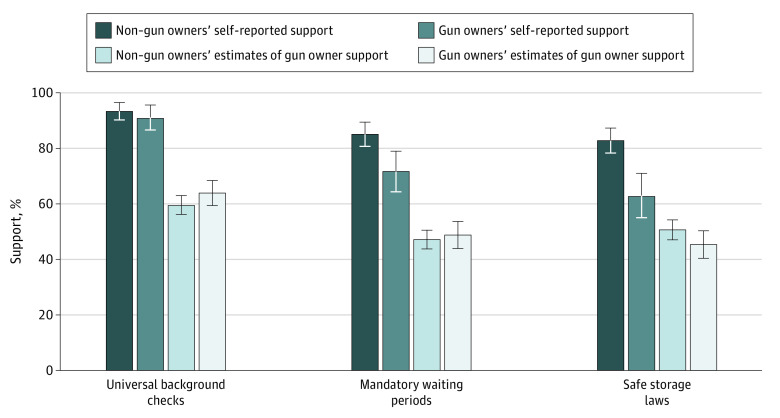
Gun Safety Policy Support Percentage of gun owners and non–gun owners who support universal background checks, mandatory waiting periods, and safe storage laws, compared with average estimates of gun owner support for these policies by gun owners and non–gun owners. Whiskers denote 95% CIs. Representative data were collected by Ipsos (market research company) from January 8 to 22, 2019. Weights applied.

Study 1 found that 63% to 91% of gun owners and 83% to 93% of non–gun owners supported key gun safety policies, including universal background checks, mandatory waiting periods, and safe storage laws, which is consistent with previous studies (Figure 1).^2^ Support for universal background checks among gun owners and non–gun owners was comparable (*F*_1,492_ = 0.63; *P* = .43; Cohen *d* = 0.14). Gun owners were less supportive than non–gun owners of mandatory waiting periods (*F*_1,492_ = 9.47; *P* = .002; Cohen *d* = 0.36) and of safe storage laws (*F*_1,492_ = 18.44; *P* < .001; Cohen *d* = 0.52). We noted that support for background checks and waiting periods was modestly higher (<5%) than in other published reports and speculate that this reflects normal variation in public opinion over time.^2^

Despite high levels of support among gun owners for these policies, both gun owners and non–gun owners underestimated support among gun owners. For universal background checks, gun owners and non–gun owners underestimated gun owner support by a 27.1 points and 31.4 points, respectively. For mandatory waiting periods, perceived support was underestimated by 23.0 points by gun owners and 24.4 points by non–gun owners. Misperceptions of gun owner support for safe storage laws were also evident, though smaller. Perceived support was underestimated by 17.6 points by gun owners and 12.3 points by non–gun owners. All differences between perceived and actual gun owner support were statistically significant (eTables 1 and 2 in the Supplement). There were no significant differences between gun owners and non–gun owners on any of these perceived support measures.

## Study 2

### Methods

Study 2, the student sample, was conducted from August 27 to October 17, 2019. Our second study was an experiment designed to test the hypothesis that correcting misperceptions about gun owners’ support for gun safety policy would be associated with an increase in participants’ policy support and would encourage them to disclose their support publicly. Participants were recruited from a participant pool composed of students enrolled in large introductory classes at the authors’ university. A total of 354 participants consented to and then completed the study (a 100% completion rate). We conducted a 2 (corrective information vs no corrective information) × 2 (system changeable vs system not changeable) laboratory experiment in which college students, most of whom did not own guns, were randomly assigned to 1 of 4 conditions. The conditions were identical except for the wording of a statement describing the 2018 mass shooting at Marjory Stoneman Douglas High School. The corrective-information conditions included published polling data highlighting the widespread support among gun owners for universal background checks (85%) and mandatory waiting periods (77%).^2,4^ We omitted this information from the no corrective information conditions. The manipulation of the perceived potential for systemic change described the policy response to the shooting. Statements in the system changeable conditions noted that Florida’s state legislature responded by passing its Public Safety Act,^27^ which broadened firearm resrictions and implemented a mandatory waiting period. The system not changeable conditions emphasized the lack of policy change at the federal level (eAppendixes 2 and 3 in the Supplement).

Following exposure to the manipulation, participants answered questions identical to those used in Study 1 for 2 gun safety issues. We excluded safe storage laws, because the system changeability manipulation referred to the Florida Public Safety Act, which did not include provisions related to safe gun storage. The survey also included a measure related to the strength of participants’ public disclosure of their support for the policies: “If you were to publicly share your views on the following gun policies, how would you describe your position to others?” Response options mirrored those used when soliciting their private support for the policies (1 = strongly opposed to 6 = strongly supportive) (eAppendix 1 in the Supplement).

Last, the study included a pair of behavioral measures. We invited participants to sign gun safety petitions directed at the US president, members of Congress, and the governor of Ohio. We then created a dichotomous variable indicating whether participants chose to sign any of them. After debriefing at the end of the study, the researcher presented participants with a box filled with stickers that read, “I support universal background checks,” and asked if they wanted any. We recorded the number of stickers each participant took and constructed a dichotomous measure indicating whether a participant took any stickers.

The study received approval from the Ohio State University institutional review board and was preregistered.^30^ Before participation, students were informed that they would be presented with information about a public policy issue and would be asked questions about it. Participants were also invited to contact the principal investigator with questions about the study before consenting, but none did. All participants provided consent via an electronic form before completing the study. Statistical analyses were conducted from May 27 to October 1, 2020, using Stata/IC, version 14.2 (StataCorp LLC) and the coefplot package (Ben Jann).^31^ *P* values were calculated using independent samples *t* test, factorial analysis of variance, and Poisson regression. All *P* values were 2 sided, and a *P* value of less than .05 was considered significant.

### Results

Study 2 had a total of 354 participants. Compared with the general population, this student sample was disproportionately young (mean [SD] age, 20.0 [2.3] years), female (232 [65.9%]), and racially identified as Asian (100 [28.3%] Asian and 37 [10.5%] Black participants) (Table). As this was a convenience sample, we have no reason to believe that it was representative of the larger student body. Furthermore, very few participants owned a gun. Nonetheless, the study allows us to test our prediction among a group that is broadly supportive of gun safety policies.

Study 2 found that exposure to corrective information was associated with a small increase in support for 2 gun safety policies of between 4% and 7%, both in terms of participants’ privately held beliefs and the beliefs they would be willing to share publicly. Manipulation checks demonstrated that the opinion-climate corrective information and system changeability manipulations were successful. An independent samples *t* test indicated that, compared with those not exposed to corrective information, participants exposed to corrective information tended to provide higher (that is, more accurate) estimates of gun owner support for universal background checks (*t*_352_ = 9.49; *P* < .001; Cohen *d* = 1.01) and for mandatory waiting periods (*t*_352_ = 12.03; *P* < .001; Cohen *d* = 1.28) (eFigure 1 in the Supplement). To assess the system changeability manipulation, participants were asked after exposure to the stimuli “To what extent do you believe that change is possible with gun policy in the United States?” (1 = not at all to 7 = very much so). Participants assigned to the system changeability conditions provided significantly higher scores than those assigned the system not changeable conditions (*t*_352_ = 3.42; *P* < .001; Cohen *d* = 0.36) (eFigure 2 in the Supplement).

**Figure 2.**
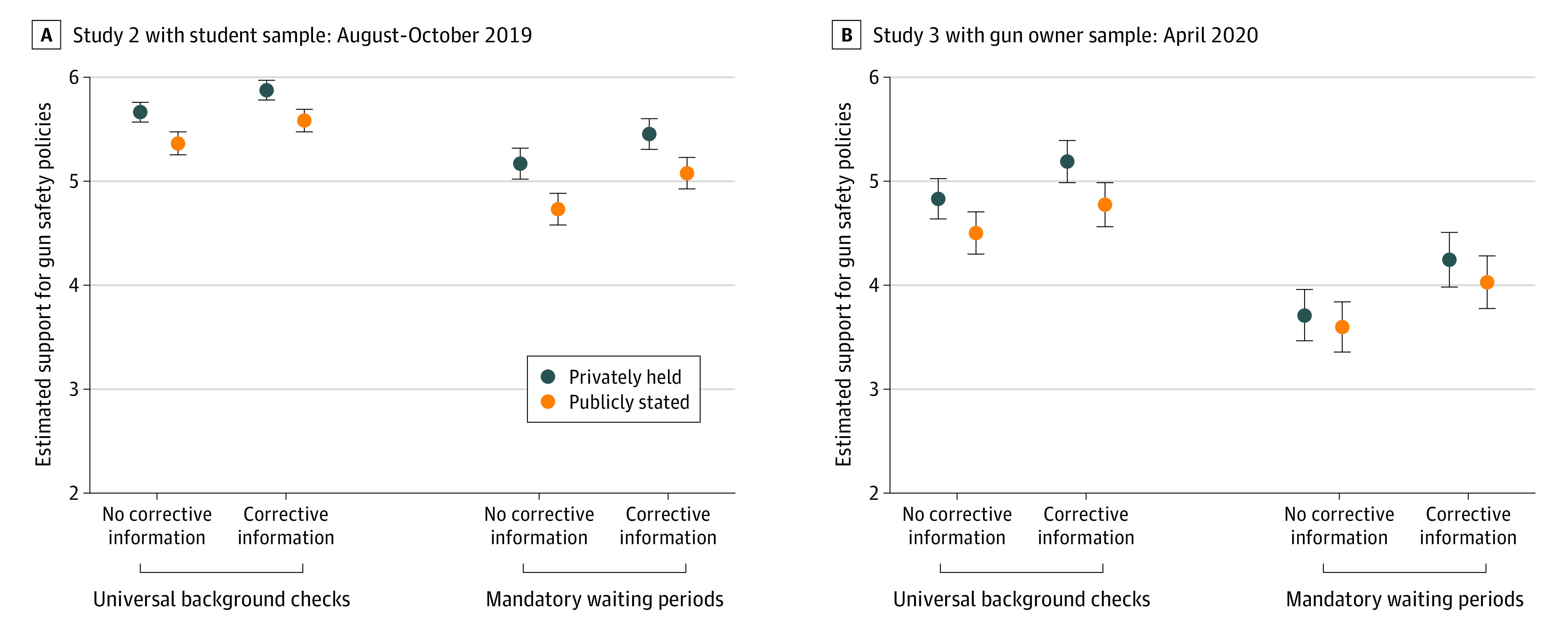
Estimated Marginal Means for Privately Held and Publicly Stated Gun Safety Policy Support A, Study 2 with student sample, surveyed from August 27 to October 17, 2019. B, Study 3 with gun owner sample, surveyed from April 15 to 21, 2020. Corrective information is presented for both surveys. Whiskers denote 95% CIs.

We used a series of factorial analyses of variance to test our hypotheses about gun attitudes. Providing corrective information about the opinion climate had a significant association with participants’ support for universal background checks (*F*_1, 351_ = 9.64; *P* = .002; η_p_^2^ = 0.03) and mandatory waiting periods (*F*_1, 351_ = 7.13; *P* = .01; η_p_^2^ = 0.02). Estimated marginal means were higher for participants exposed to corrective information than for participants not exposed to corrective information (Figure 2A). Corrective information also had a significant association on how supportive participants said they would be when sharing their views publicly about universal background checks (*F*_1, 351_ = 7.96, *P* = .005, η_p_^2^ = 0.02) and about mandatory waiting periods (*F*_1, 351_ = 10.1, *P* = .002, η_p_^2^ = 0.03). Estimated marginal means were higher for those exposed to corrective information than for those not exposed to corrective information (Figure 2A). Neither private nor public levels of support were conditioned on system changeability for either issue.

We estimated relative risk of the 2 behavioral outcomes using a series of Poisson regression models with robust error variance. Corrective information had no association on whether participants signed any petitions (relative risk = 0.98; 95% CI, 0.89-1.08; *P* = .72) or took any stickers (relative risk = 1.00; 95% CI, 0.92-1.09; *P* = .97).

## Study 3

### Methods

The third study sought to replicate the findings from Study 2 using a nationally representative sample of gun owners. Study 3 consisted of the same design and materials as Study 2, with a few key alterations. Data collection was administered by the marketing research company YouGov from April 15 to 21, 2020. YouGov maintains a large opt-in panel of participants and uses a sample matching methodology to construct representative samples. This matching is done in 2 steps. First, a random sample is drawn from census data corresponding to the target population. Second, for each member of this sample, the company selects panelists who match sample members’ demographic characteristics. For this study, the company recruited 6503 US adults from its panel and screened the group based on personal gun ownership, identifying 727 gun owners. These individuals were then matched down to form a total sample of 400 gun owners who then participated in the study. The company also provided poststratification weights, which we used in all computations (eAppendixes 2 and 3 in the Supplement).

All data collection was conducted online. Like Study 2, this was a 2 (corrective information vs no corrective information) × 2 (system changeable vs system not changeable) experiment in which participants were randomly assigned to 1 of 4 conditions. The study used the same stimuli and self-report measures, but behavioral measures were different. First, we only invited participants to sign a single gun safety petition directed at Congress. Second, instead of offering stickers to participants, we provided a $20 cash incentive and invited participants to donate any portion of this payment to the Sandy Hook Promise, an organization devoted to gun safety. We provided a short description of the charity, including its mission to lobby for universal background checks (eAppendixes 2 and 3 in the Supplement).

This study received approval from the Ohio State University institutional review board and was preregistered.^32^ Panelists were informed that they would be presented with information about a public policy issue and would be asked questions about it. All participants provided consent via an electronic form before completing the study. All statistical tests, including proportions, were computed using survey weights. Statistical analyses were conducted from May 26 to October 16, 2020, using Stata/IC, version 14.2 (StataCorp LLC) and the coefplot^31^ (Ben Jann) and admetan^33^ (David Fisher) packages. *P* values were calculated using analysis of variance, factorial analysis of variance, and Poisson regression. All *P* values were 2 sided, and a *P* value of less than 0.05 was considered significant.

### Results

Study 3 consisted of 400 participants (mean [SD] age, 52.1 [16.4] years; 212.7 men [53.2%]; 295.5 White participants [73.9%]). The 400 participants in Study 3 were demographically diverse; however, because this study was limited to gun owners, there were some notable differences from the general US population (Table). Participants tended to be older (mean [SD] age, 52.1 [16.4] years) and included fewer women (187.3 [46.8%]) and more White participants (295.5 [73.9%] White, 44.5 [11.1%] Hispanic, and 32.4 [8.1%] Black participants). They were also more likely to have only finished high school (136.7 respondents [34.2%]) and to live in the American South (203.7 respondents [50.9%]).

Study 3 found that exposure to corrective information was associated with a small increase in support for 2 gun safety policies of between 6% and 15%, both in terms of participants’ privately held beliefs and the beliefs they would be willing to share publicly. Manipulation checks indicated that all manipulations were successful. An analysis of variance indicated that, compared with those not exposed to corrective information, participants exposed to corrective information provided higher (that is, more accurate) estimates of gun owner support for universal background checks (*F*_1, 396_ = 27.46; *P* < .001; η_p_^2^ = 0.06) and for mandatory waiting periods (*F*_1, 396_ = 50.72; *P* < .001; η_p_^2^ = 0.11) (eFigure 3 in the Supplement). Similarly, participants assigned to the system changeable conditions were significantly more likely to describe political change as possible than those assigned the system not changeable conditions (*F*_1, 398_ = 11.36; *P* < .001; η_p_^2^ = 0.03) (eFigure 4 in the Supplement).

A series of factorial analyses of variance confirms that corrective information had a significant association with participants’ support for both universal background checks (*F*_1, 397_ = 6.31; *P* = .01; η_p_^2^ = 0.02) and mandatory waiting periods (*F*_1, 396_ = 8.44; *P* = .004; η_p_^2^ = 0.02). Estimated marginal means were higher for participants exposed to corrective information than for participants not exposed to corrective information (Figure 2B). Corrective information did not have a significant association with how supportive participants said they would be when sharing their views publicly for universal background checks (*F*_1, 397_ = 3.33; *P* = .07; η_p_^2^ = 0.01), but the association with mandatory waiting periods was significant (*F*_1, 397_ = 5.86; *P* = .02; η_p_^2^ = 0.02). Again, estimated marginal means are higher for those exposed to corrective information than those not exposed to corrective information (Figure 2B). None of these relationships were conditioned on system changeability.

**Figure 3.**
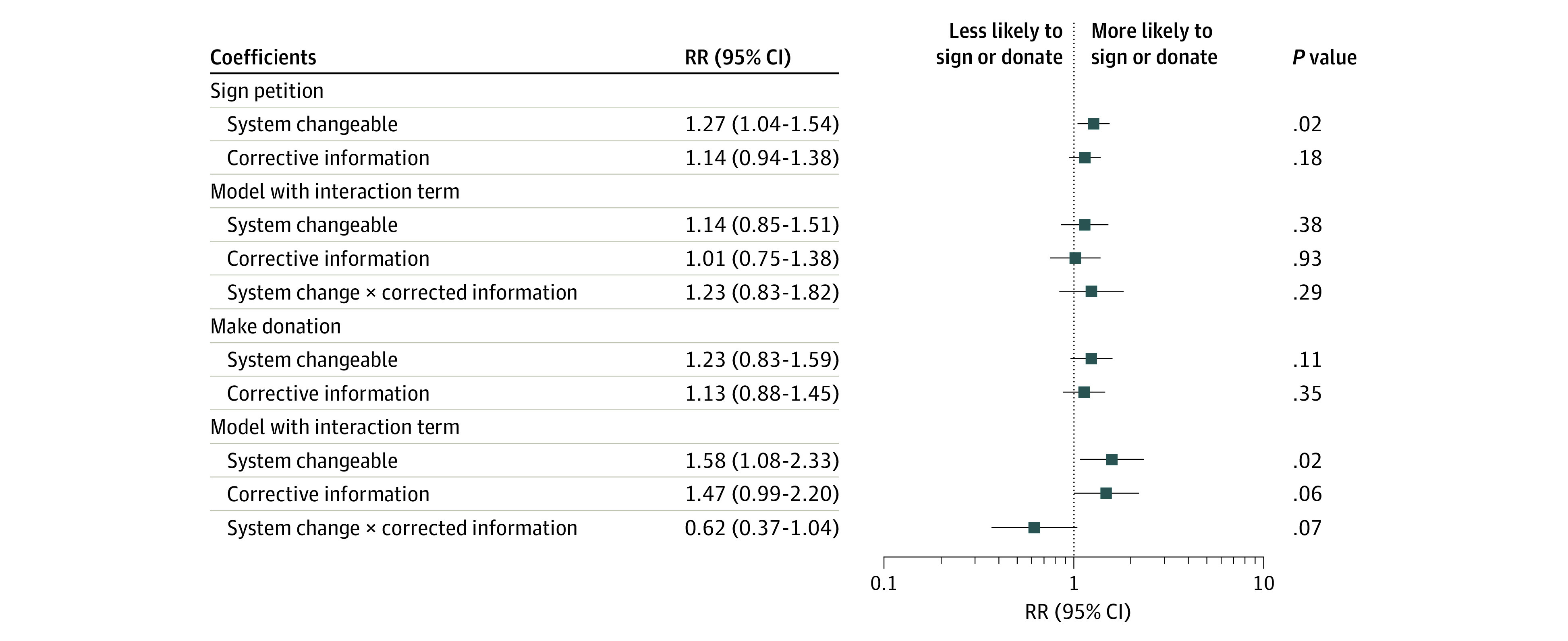
Comparing Coefficients of System Changeability and Corrective Information on 2 Policy-Support Behaviors For Study 3 only, conducted from April 15 to 21, 2020. RR indicates relative risk.

Corrective information did not significantly associate with participants’ observed behaviors. We estimated relative risk of the 2 behavioral outcomes using a series of Poisson regression models with robust error variance (Figure 3). We found that the system changeability message was associated with the participants’ likelihood of signing the petition, but corrective information had no relationship. Neither system changeability nor corrective information has a significant association with donating.

## Discussion

The representative survey data from Study 1 suggest that gun owners and non–gun owners overwhelmingly support universal background checks, mandatory waiting periods, and safe storage laws. Furthermore, both groups underestimated the level of support for these policies among the gun-owning public. The 2 follow-up experiments (Studies 2 and 3) found that providing accurate information about the opinion climate was associated with increases in private and public expression of support for gun safety policies among both gun owners and non–gun owners.

We found little evidence, however, that information about the opinion climate was associated with behavior. Individuals’ willingness to take stickers expressing their policy support, to sign a petition advocating for gun safety policies, and to make a donation in support of gun safety were all unchanged following exposure to this information.

### Limitations

One important limitation of the research is that our associations, though statistically significant, were small in magnitude. However, small associations can still be meaningful when considered in context.^34^ Even modest increases in individuals’ willingness to voice support for gun safety policies could have important consequences when multiplied across the population. Small associations can also be important at the local level, where policy change is often more politically feasible.

The nonrepresentative student sample used in Study 2 is another limitation, especially in contrast to the representative samples used in Studies 1 and 3. Study 2 participants were generally young, disproportionately women, and rarely owned guns. However, the consistency observed across the results of Studies 2 and 3 is striking, suggesting that the findings are robust in the face of significant demographic differences.

These experiments do not take into consideration participants’ prior attitudes about gun safety policies. It is possible that individuals who oppose them would question the accuracy of polling data, undermining message effects. Alternatively, learning that they are in the minority might cause some participants to entrench their position.^21^ This might be especially likely for those who hold extreme anti–gun policy attitudes and for those who hold those attitudes with high confidence.^35^ Receptivity to corrective information could also be influenced by its source. Corrective information is often more effective when it comes from a credible source, especially one associated with the in-group.^36,37^

Finally, drawing on the common in-group identity model, we speculate that accurate information about public opinion might elicit recategorization of gun owners by non–gun owners, thereby reducing intergroup distinctions.^38^ This can, in turn, reduce intergroup bias, as group members’ cognitive representations change from 2 groups to 1—that is, from an “us” vs “them” to an inclusive “we.”^38^ We cannot test this idea with these data, but future research should examine whether recategorization helps explain why correcting perceptions of public opinion is effective.

## Conclusions

The findings of this 3-part study provide additional evidence regarding a perceived opinion climate’s association with policy attitudes and suggest more practically that misperceptions about gun owners’ support for gun safety policy may constrain public support for these policies. These results provide insight for policy makers and advocates and suggest that correcting misperceived opinion climates may be a useful strategy for encouraging both private and public support for gun safety policies.

## Notes

Supplement. **eAppendix 1.** Data Collection Details **eAppendix 2.** Study Protocol for Study 1 **eAppendix 3.** Study Protocol for Studies 2 and 3 **eFigure 1.** Corrective Information Manipulation Check, Study 2 **eFigure 2.** System Changeability Manipulation Check, Study 2 **eFigure 3.** Corrective Information Manipulation Check, Study 3 **eFigure 4.** System Changeability Manipulation Check, Study 3 **eTable 1.** Gun Owner and Non-Owners Actual and Estimated Support for Gun Safety Policies **eTable 2.** Significance of Underestimating Gun Owner Support for Gun Safety Policies **eReferences**
