# Supplementary material for: Public Opinion Perceptions, Private Support, and Public Actions of US Adults Regarding Gun Safety Policy
Source: JAMA Netw Open. 2020 Dec 22;3(12):e2029571. doi: 10.1001/jamanetworkopen.2020.29571 (PMC7756237; doi:10.1001/jamanetworkopen.2020.29571)
Supplement: Supplement. — eAppendix 1. Data Collection Details eAppendix 2. Study Protocol for Study 1 eAppendix 3. Study Protocol for Studies 2 and 3 eFigure 1. Corrective Information Manipulation Check, Study 2 eFigure 2. System Changeability Manipulation Check, Study 2 eFigure 3. Corrective Information Manipulation Check, Study 3 eFigure 4. System Changeability Manipulation Check, Study 3 eTable 1. Gun Owner and Non-Owners Actual and Estimated Support for Gun Safety Policies eTable 2. Significance of Underestimating Gun Owner Support for Gun Safety Policies eReferences [file jamanetwopen-e2029571-s001.pdf]

Supplementary Online Content

Dixon G, Garrett K, Susmann M, Bushman BJ. Public opinion perceptions, private support, and public actions of US adults regarding gun safety policy. *JAMA Netw Open*. 2020;3(12):e2029571. doi:10.1001/jamanetworkopen.2020.29571

eAppendix 1. Data Collection Details

eAppendix 2. Study Protocol for Study 1

eAppendix 3. Study Protocol for Studies 2 and 3

eFigure 1. Corrective Information Manipulation Check, Study 2

eFigure 2. System Changeability Manipulation Check, Study 2

eFigure 3. Corrective Information Manipulation Check, Study 3

eFigure 4. System Changeability Manipulation Check, Study 3

eTable 1. Gun Owner and Non-Owners Actual and Estimated Support for Gun Safety Policies

eTable 2. Significance of Underestimating Gun Owner Support for Gun Safety Policies

eReferences

This supplementary material has been provided by the authors to give readers additional information about their work.

© 2020 Dixon G et al. *JAMA Network Open.*

eAppendix 1. Data Collection Details

Study 1: Ipsos

KnowledgePanel is the largest online panel that relies on probability-based sampling techniques for recruitment; hence, it is the largest national sampling frame from which fully representative samples can be generated to produce statistically valid inferences for study populations. It provides samples with the highest level of representativeness available in online research for measurement of public opinions, attitudes, and behaviors. Participants in the panel complete a short demographic survey, which is used to generate weights. The weighting is based on the U.S. Census Bureau's Current Population Survey. All panel members are provided privacy and confidentiality protections. Once panel members complete the survey, they become eligible for selection for surveys. Once assigned to a survey, members receive a notification email letting them know there is a new survey available for them to complete. This email notification contains a link that sends them to the survey. No login name or password is required.

Study 2: C-REP

Communication Research Experience Program (C-REP) provides faculty and graduate students in The Ohio State University School of Communication access to a large pool of undergraduate research participants. C-REP is required for students enrolled communication introductory courses and is optional for other courses.

Study 3: YouGov

YouGov recruited a representative sample of 6,503 US adults and screened those respondents for personal gun ownership. YouGov interviewed 727 qualified respondents who were then matched down to a total sample of 400 gun owners to produce the final dataset. The respondents were matched using a "census clicks" approach, where the full sample of general population are matched to a sampling frame on gender, age, race, and education. The frame was constructed by stratified sampling from the sample of general population from the 2017 American Community Survey (ACS), with selection within strata by weighted sampling with replacements (using the person weights on the public use file). The matched general population cases were weighted to the sampling frame using propensity scores. The matched cases and the frame were combined, and a logistic regression was estimated for inclusion in the frame. The propensity score function included age, gender, race/ethnicity, years of education, and region. The propensity scores were grouped into deciles of the estimated propensity score in the frame and post-stratified according to these deciles. The weights were then post-stratified on 2016 Presidential vote choice, and a four-way stratification of gender, age (4-categories), race (4- categories), and education (4-categories). The sample was then subset on gun owners, and the weights were trimmed to a maximum value of 7 and centered around 1.

© 2020 Dixon G et al. *JAMA Network Open.*

Question wording: Study 1
This is the last section of the survey. Our goal here is to better understand your attitudes toward gun control policy.

OWNGUN [S]
Do you currently own a firearm?
1. Yes
2. No

POLSUP [S Grid]
To what extent do you support or oppose the following policies?
Statements in row (randomize and record response order):
a. Requiring a background check system for all gun sales to make sure a purchaser is not legally prohibited from having a gun
b. Imposing a federal mandatory waiting period on all gun purchases, so that everyone who purchases a gun must wait a certain number of days before taking the gun home
c. Requiring by law that a person lock up the guns in their home when not in use to prevent handling by children or teenagers without adult supervision

Answers in column:
1. Strongly opposed
2. Opposed
3. Somewhat opposed
4. Somewhat supportive
5. Supportive
6. Strongly supportive

OWNERS [N, prompt once]
What percent of American gun owners do you think support the following policies?
(Small textbox followed by "% of gun owners support", and accepting values between 0 to 100)
Statements in row (use order assigned in POLSUP):
a. Requiring a background check system for all gun sales to make sure a purchaser is not legally prohibited from having a gun
b. Imposing a federal mandatory waiting period on all gun purchases, so that everyone who purchases a gun must wait a certain number of days before taking the gun home
c. Requiring by law that a person lock up the guns in their home when not in use to prevent handling by children or teenagers without adult supervision
Answers in column:
1. [NUMBERBOX, RANGE 0 - 100] % of gun owners support this

Question wording: Studies 2 and 3
To what extent do you believe that change is possible with gun policy in the United States? (1 = *not at all*, 7 = *very much so*).

To what extent do you support or oppose the following policies? (1 = *Strongly opposed*, 2 = *Opposed*, 3 = *Somewhat opposed*, 4 = *Somewhat supportive*, 5 = *Supportive*, 6 = *Strongly supportive*)

© 2020 Dixon G et al. *JAMA Network Open.*

a. Requiring a background check system for all gun sales to make sure a purchaser is not legally prohibited from having a gun.
b. Imposing a federal mandatory waiting period on all gun purchases, so that everyone who purchases a gun must wait a certain number of days before taking the gun home

We'd now like to ask again how much support you think American gun owners give to certain gun policies. What percent of American gun owners do you think support the following policies? (0% to 100%)

a. Requiring a background check system for all gun sales to make sure a purchaser is not legally prohibited from having a gun
b. Imposing a federal mandatory waiting period on all gun purchases, so that everyone who purchases a gun must wait a certain number of days before taking the gun home

If you were to publicly share your views on the following gun policies, how would you describe your position to others? (1 = *Strongly opposed*, 2 = *Opposed*, 3 = *Somewhat opposed*, 4 = *Somewhat supportive*, 5 = *Supportive*, 6 = *Strongly supportive*)

a. Requiring a background check system for all gun sales to make sure a purchaser is not legally prohibited from having a gun
b. Imposing a federal mandatory waiting period on all gun purchases, so that everyone who purchases a gun must wait a certain number of days before taking the gun home?

The following page contains a petition sent to government officials urging them to support changes in gun background checks so that all gun purchases (including private sales) are subject to background checks. Please sign the petition only if you want to.

> Tell Congress You Want Universal Background Checks
>
> TO: MEMBERS OF CONGRESS
>
> FROM: [YOUR NAME]
>
> Everyone who buys a gun should be vetted. We demand you do what needs to be done to protect our citizens from gun violence and pass this critical legislation.
>
> Sincerely,
> [Your name]

Do you wish to sign your name to this petition? By clicking yes, your name will be automatically submitted to the online petition. (1 = *Yes*, 2 = *No*)

© 2020 Dixon G et al. *JAMA Network Open.*

[Study 2 only]

> Tell Governor Mike DeWine You Want Universal Background Checks
> To: Governor Mike DeWine
> From: [Your name]
> Everyone who buys a gun should be vetted. We demand you do what needs to be done to protect our citizens from gun violence by supporting universal background checks.
> Sincerely,
> [Your name]

Do you wish to sign your name to this petition? By clicking yes, your name will be automatically submitted to the online petition. (1 = *Yes*, 2 = *No*)
[study 2 only]

> Tell President Trump You Want Universal Background Checks
> To: President Trump
> From: [Your name]
> Everyone who buys a gun should be vetted. We demand you do what needs to be done to protect our citizens from gun violence by supporting universal background checks.
> Sincerely,
> [Your name]

Do you wish to sign your name to this petition? By clicking yes, your name will be automatically submitted to the online petition. (1 = *Yes*, 2 = *No*)

[Study 3 only] Finally, you have the opportunity to donate some or all of your $20 reward to The Sandy Hook Promise. Founded by two parents of Sandy Hook victims, The Sandy Hook Promise is a moderate, above-the-politics organization that supports sensible program and policy solutions that address the “human side” of gun violence by preventing individuals from ever getting to the point of picking up a firearm to hurt themselves or others.
The organization currently lobbies for Congress to pass universal background checks.

How much of your $20 reward would you like to donate? ($0 to $20 sliding scale in $1 increments)

Control variables
Age (2020 – birth year)
What do you consider yourself? (American Indian or Alaska Native, Black or African American, White, Asian, Native Hawaiian or Pacific Islander, other)
Please select your sex (male, female, prefer not to answer)
In general, I would describe my political views as _____ (1= very conservative, 2= conservative, 3 = slightly conservative, 4 = moderate; 5 = slightly liberal, 6 = liberal, 7 = very liberal)
Do you currently own a firearm? (1 = *Yes*, 2 = *No*)

© 2020 Dixon G et al. *JAMA Network Open.*

Stimuli: Studies 2 and 3

"Next, we want to give you more information about a particular policy. After reading, you'll be asked your views of this policy."

[Randomly assigned]

System change/No Gun consensus information

On February 14, 2018, a gunman opened fire at Marjory Stoneman Douglas High School in Parkland, Florida, killing 17 students and staff members and injuring 17 others. Following the massacre, many Parkland student survivors and members of the public began lobbying for legislative action on gun violence. Due to public demand for changes in gun policy, lawmakers in Florida's state legislature passed bipartisan legislation that established mandatory waiting periods and strengthened background checks for gun buyers. This shows that changes to gun policy is possible in the wake of mass shootings.

System change/Gun consensus information

On February 14, 2018, a gunman opened fire at Marjory Stoneman Douglas High School in Parkland, Florida, killing 17 students and staff members and injuring 17 others. Following the massacre, many Parkland student survivors and members of the public began lobbying for legislative action on gun violence. A majority of American gun owners support such actions. For instance, a recent national poll found that 85% of gun owners support requiring background checks on all gun sales, and 77% support a federal mandatory waiting period on all gun purchases. Due to public demand for changes in gun policy, Florida's state legislature passed bipartisan legislation that established mandatory waiting periods and strengthened background checks for gun buyers. This shows that changes to gun policy is possible in the wake of mass shootings.

No System change/Gun consensus information

On February 14, 2018, a gunman opened fire at Marjory Stoneman Douglas High School in Parkland, Florida, killing 17 students and staff members and injuring 17 others. Following the massacre, many Parkland student survivors and members of the public began lobbying for legislative action on gun violence. A majority of American gun owners support such actions. For instance, a recent national poll found that 85% of gun owners support requiring background checks on all gun sales, and 77% support a federal mandatory waiting period on all gun purchases. Despite public demand for changes in gun policy, lawmakers in Washington have failed to pass legislation that establishes mandatory waiting periods and strengthens background checks for gun buyers. This shows that changes to gun policy is difficult even in the wake of mass shootings.

No System change/ No Gun consensus information

On February 14, 2018, a gunman opened fire at Marjory Stoneman Douglas High School in Parkland, Florida, killing 17 students and staff members and injuring 17 others. Following the massacre, many Parkland student survivors and members of the public began lobbying for legislative action on gun violence. Despite public demand for changes in gun policy, lawmakers in Washington have failed to pass legislation that establishes mandatory waiting periods and strengthens background checks for gun buyers. This shows that changes to gun policy is difficult even in the wake of mass shootings.

© 2020 Dixon G et al. *JAMA Network Open.*

eAppendix 2. Study Protocol for Study 1

Note: This was an omnibus survey. Descriptions of work conducted by other researchers has been omitted.

I. Objectives
This is an omnibus survey sponsored by the School of Communication. It is a collaboration between three teams of researchers, each with their own objectives. This protocol describes each team in its own section.
Dixon: Our team wants to assess the extent to which Americans, both those who own guns and those who do not, underestimate support for gun-control regulation among gun owners, and how this misperception may impact behaviors and attitudes toward gun policy.

II. Background and Rationale
Dixon: The American public is often portrayed as highly polarized on gun policy (Barry et al., 2018). Yet, research has consistently found that majorities of both gun owners and non-owners support key gun control proposals, including expanded background checks, mandatory waiting periods, and required child safety locks (Barry et al., 2018; Igielnik & Brown, 2017; Quinnipiac, 2018; Parker et al., 2017). Despite these similarities, the two groups differ in their approaches to policy advocacy. We aim to demonstrate that gun owners are less likely than non-owners to advocate for, and to vote in favor of, their preferred gun policies. Further, we seek to explain this behavior by showing that gun owners who support stricter gun laws perceive their views as atypical of other gun-owners. This project, therefore, will document how misperceived opinion climates and party cues may negatively affect gun owners' public advocacy, actions, and attitudes toward the policies they privately support.

III. Procedures
A. Research Design
We propose to conduct a 20-minute online survey. Data collection is anticipated to take about three weeks.

B. Sample
Respondents will be recruited from a representative online panel maintained by the GfK Group. This company maintains a panel composed of U.S. adults who have volunteered to take surveys. We are seeking approximately 500 respondents.

C. Measurement / Instrumentation
The study uses established survey measures when they are available. In instances where preexisting measures are unavailable, we have created our own.

D. Detailed study procedures
Subjects will be recruited by GfK from the KnowledgePanel, a representative online panel that the company created and maintains. Randomly selected individuals who have already volunteered to participate in the survey panel will receive an email invitation with an embedded, secure, and individualized link to the survey instrument.

E. Internal Validity
The study uses establish measures when possible.

F. Data Analysis
Statistical descriptives, analysis of variances tests, and regression tests will be used to analyze data. Statistical modeling software such as SPSS, Stata, and/or R will be used.

© 2020 Dixon G et al. *JAMA Network Open.*

Bibliography

Barry, C. L., Webster, D. W., Stone, E., Crifasi, C. K., Vernick, J. S., & McGinty, E. E. (2018). Public Support for Gun Violence Prevention Policies Among Gun Owners and Non–Gun Owners in 2017. American journal of public health, 108(7), 878-881

Igielnik, R. & Brown, A. (2017). Key takeaways on Americans' views of guns and gun ownership. Pew Research Center. Retrieved from: http://www.pewresearch.org/fact tank/2017/06/22/key-takeaways-on-americans-views-of-guns-and-gun-ownership

Parker, K., Horowitz, J., Igielnik, R., Oliphant, B., & Brown, A. (2017). America's complex relationship with guns. Chapter 5. Views on guns. Retrieved from: http://www.pewsocialtrends.org/2017/06/22/views-on-gun-policy/

Quinnipiac University Poll (2018). U.S. Support For Gun Control Tops 2-1, Highest Ever, Quinnipiac University National Poll Finds; Let Dreamers Stay, 80 Percent Of Voters Say. Retrieved from: https://poll.qu.edu/national/release-detail?ReleaseID=2521

© 2020 Dixon G et al. *JAMA Network Open.*

eAppendix 3. Study Protocol for Studies 2 and 3

I. Objectives

The goal of this study is to examine under what conditions public consensus about gun policies influence public actions. Specifically, we explore whether public opinion information detailing gun owners' support for key gun policies can influence people's public expression of support for these policies. We also examine whether public opinion information is most effective when people perceive gun policy can be changed.

II. Background and Rationale

Gun violence is a persistent issue in the United States, with over 175,000 deaths recorded between 2012 and 2016 (CDC, 2017). Policies aimed at curbing gun violence receive wide support among the American public. Recent polls, for instance, report that a majority of gun owners and non-owners alike support expanded background checks, mandatory waiting periods, and requirements for safe storage of firearms (Barry et al., 2018; Igielnik & Brown, 2017; Quinnipiac, 2018; Parker et al., 2017a). Despite this agreement, gun owners display more apprehension toward enacting new gun laws and are more likely to vote for political candidates who reject the gun policies that they typically support (Cohn & Quealy, 2017; Parker et al., 2017). Furthermore, gun control supporters show less willingness than gun advocates to express their views, sign petitions, and contact elected officials (Drake, 2013). As a result, the American public has largely remained apathetic towards mobilizing for gun policy changes (Singh, 1998). Thus, the American public's attitudes and actions toward these policies may not necessarily reflect their private support.

One reason for this is due to misperceived norms, such as falsely perceiving that one's support for gun policies reflects the views of only their in-group, such as with non-gun owners, or atypical for their in-group, as in the case of gun owners (Miller & McFarland, 1991). Communicating consensus information could be used for correcting misperceived norms. First, correcting misperceptions by informing people their views are held by their in-group can reverse pluralistic ignorance (Geiger & Swim, 2016; Schroeder & Prentice, 1998). Highlighting a consensus does this by correcting misperceived norms, where decreasing the gap between perceived and actual normative views can be used as leverage to influence beliefs and actions (see van der Linden et al., 2015). For example, health interventions identifying actual normative behaviors of an oft misperceived norm (i.e., alcohol consumption) can influence subsequent attitudes and behavior (e.g. Haines & Spear, 1996). In work on scientific controversies, many people underestimate the degree of scientific consensus for issues like climate change and vaccines (Dixon & Clarke, 2013; Lewandowsky et al., 2013). By highlighting a consensus, estimates of scientific support are increased, which can then help shift people's personal views, attitudes, and policy support (van der Linden et al., 2015; Dixon et al., 2015). Thus, corrective consensus information can act as a "Gateway" to correcting one's personal beliefs and actions (van der Linden et al., 2015).

Indeed, in our first study, we found that both gun owners and non-owners underestimate the percent of gun owners who support key gun policies. We, therefore, propose that informing people that a consensus among gun owners exists can spur greater public expression of support for gun policies.

H1: Informing that a majority of gun owners support key gun policies results in people reporting: higher estimates of gun owner support, perceiving gun policies as more effective, greater willingness to publicly express support for policies, perceiving gun policies as more urgent, and a lesser willingness to use deception when publicly express support for policies.

We also explore whether the presence of system change information moderates the effect of consensus information. For instance, gun policy has rarely shifted at the federal level, giving the impression that change is slow, difficult, and improbable. Research has found that impressions that systems are changeable can lead people to develop negative evaluations of status quo (Johnson & Fujita, 2012). As a result, impressions that gun

© 2020 Dixon G et al. *JAMA Network Open.*

policy is changeable could lead consensus information to be more mobilizing. Thus, with consensus information, we investigate whether system change moderates the effect of consensus information

H2a: The presence of system change information will moderate the effect of consensus information on public support.

H2b: When consensus information includes system change information, it will be most effective at influencing public expressions of support.

III. Procedures

A. Research Design

The proposed study will be a 2 (consensus statement versus no consensus statement) X 2 (system changeable versus no system changeable) experiment. Each participant will be presented with a short statement about the recent school shooting at Marjory Stoneman Douglas High School in 2018. Our "changeable" manipulation will describe how in the aftermath of the shooting, Florida's state legislature passed bipartisan legislation that strengthened background checks and implemented a mandatory waiting period. The "non-changeable" manipulation will mention how the federal government has not made any changes to strengthening background checks and implementing a mandatory waiting period. The "consensus information" manipulation will include statistical polling data showing the percent of gun owners that support universal background checks and mandatory waiting periods. All manipulated content is factual. No deception is being used with this content.

They will also answer pre- and post- exposure questionnaires pertaining to key variables of interest and general demographics. Our key outcome variables are support for gun control policy, public disclosure of support for gun policy, and behaviors that express support for gun policy.

Two studies will be performed using the same design. The lab-based study will involve a student sample and its main outcome behavioral variable is the amount of gun control stickers selected. The online study involves only gun owners, and the main outcome behavioral variable is the participant's decision to donate to a gun control organization. Aside from these differences, the two studies are identical.

B. Sample

In our first in-lab study, participants will be recruited from research pools from the authors' institution.

We plan on recruiting roughly 400 participants starting Autumn 2019. There will be no social pressures exerted online regarding participation. These numbers were selected in order to make sure that experimental cells are large enough while staying in budget and accounting for participants who are not as engaged in the study since it is completed online on their own time. Indeed, a large sample size will also benefit us given the somewhat small effect sizes typical in research on the effects of exposure to brief media messages.

A second study will consist of only gun owners with the same sample size.

C. Data Analysis

Data will be analyzed using statistical programs such as SPSS. Appropriate statistical analyses (e.g. regression models, ANOVAs, t-tests) will be employed to examine the effects.

© 2020 Dixon G et al. *JAMA Network Open.*

eFigure 1. Corrective Information Manipulation Check, Study 2

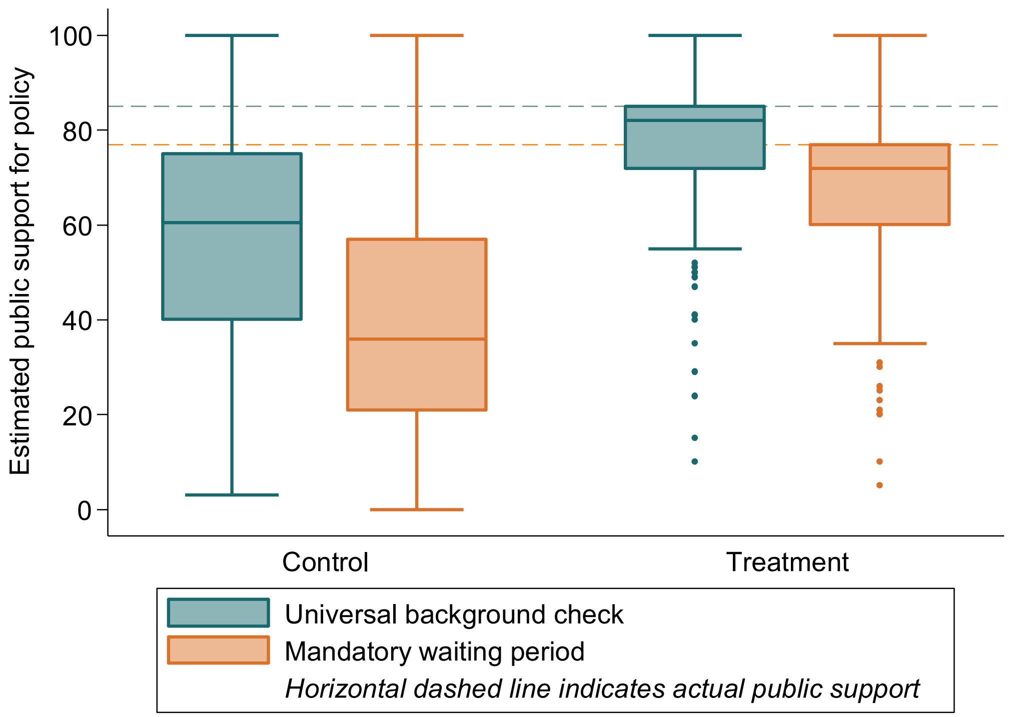

© 2020 Dixon G et al. *JAMA Network Open.*

eFigure 2. System Changeability Manipulation Check, Study 2

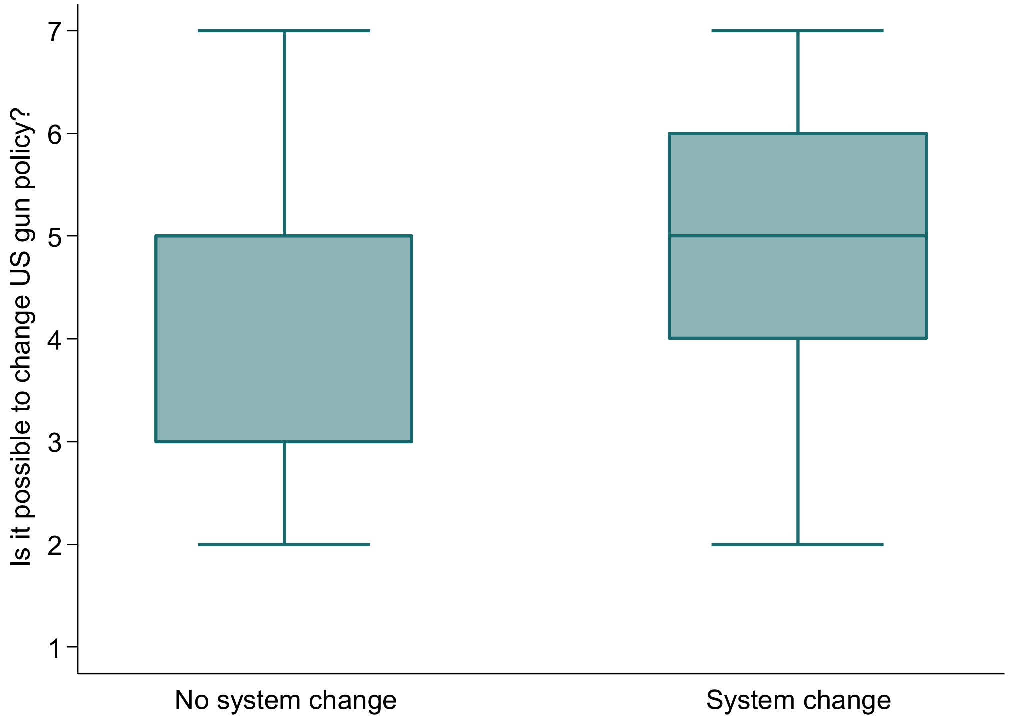

© 2020 Dixon G et al. *JAMA Network Open.*

eFigure 3. Corrective Information Manipulation Check, Study 3

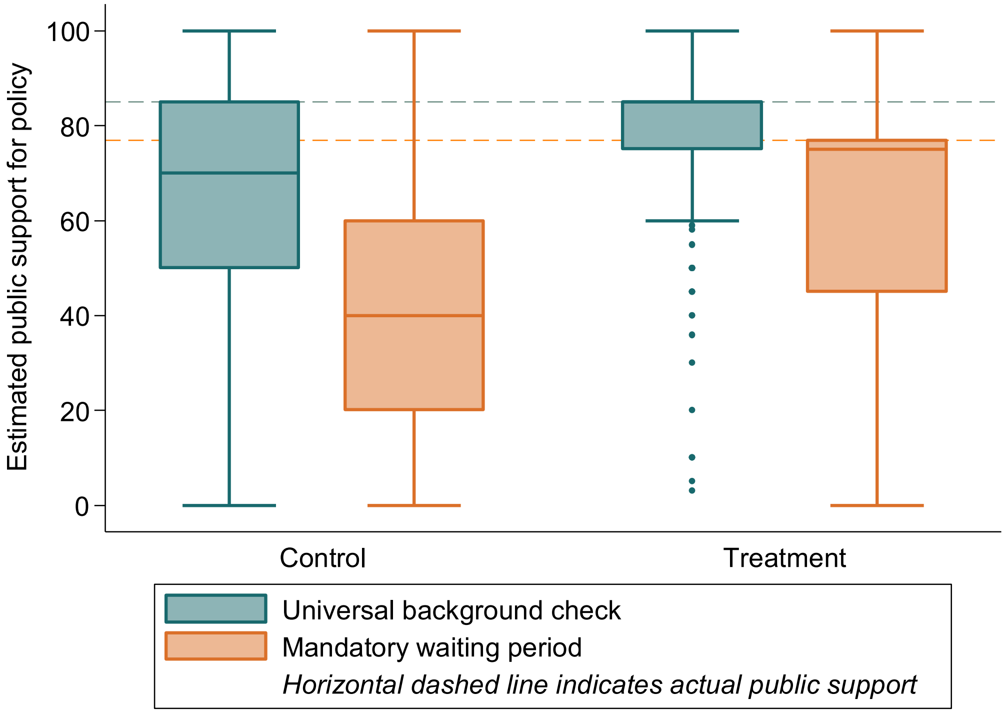

© 2020 Dixon G et al. *JAMA Network Open.*

eFigure 4. System Changeability Manipulation Check, Study 3

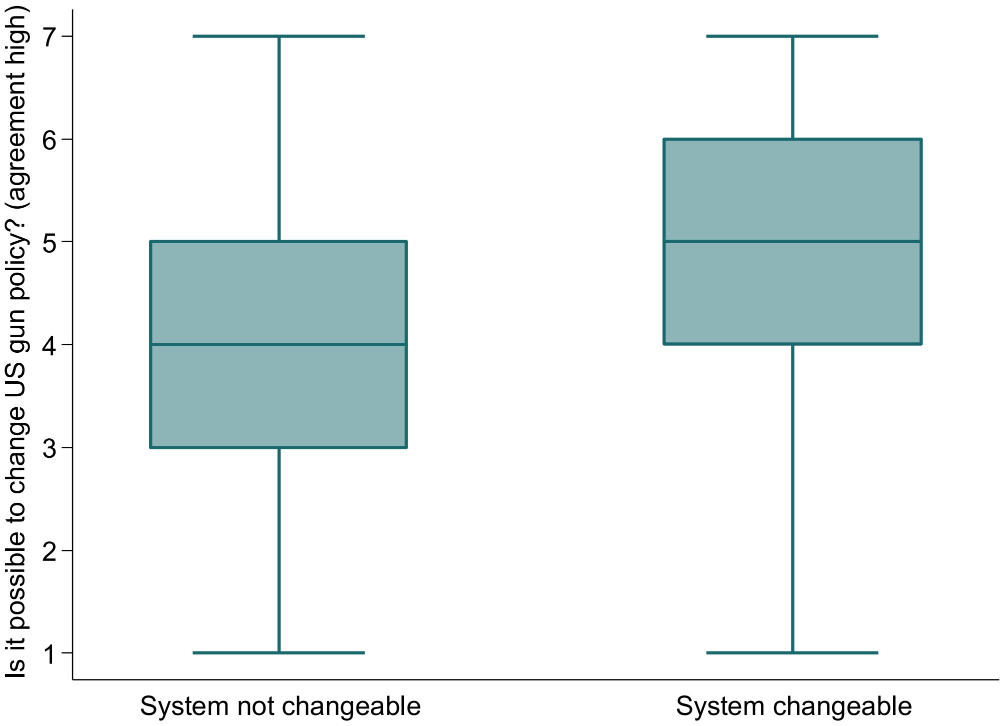

© 2020 Dixon G et al. *JAMA Network Open.*

eTable 1. Gun Owner and Non-Owners Actual and Estimated Support for Gun Safety Policies

	Universal background checks	Mandatory waiting periods	Safe storage laws
	Mean (SE)	Mean (SE)	Mean (SE)
Non-gun owner's self-reported support	93% (2%)	85% (2%)	83% (2%)
Gun owners' self-reported support	91% (2%)	72% (4%)	63% (4%)
Non-gun owners' estimates of gun owner support	60% (2%)	47% (2%)	51% (2%)
Gun owners' estimates of gun owner support	64% (2%)	49% (3%)	45% (3%)

© 2020 Dixon G et al. *JAMA Network Open.*

eTable 2. Significance of Underestimating Gun Owner Support for Gun Safety Policies

	Gun owners estimates (row 4 vs. row 2)	Non-gun owners estimates (row 3 vs. row 2)	Gun owner and non-owner estimates (row 3 vs. row 4)
Universal background checks	z (491) = 10.20, p < .0001	z (491) = 11.44, p < .0001	F (1,490) = 2.16, p = .14
Mandatory waiting periods	z (491) = 7.36, p < .0001	z (491) = 7.80, p < .0001	F (1,490) = .23, p = .63
Safe storage laws	z (491) = 3.91, p < .0001	z (491) = 5.55, p < .0001	F (1,490) = 2.88, p = .09

© 2020 Dixon G et al. *JAMA Network Open.*

eReferences

Asch, S. E. (1951). Effects of group pressure upon the modification and distortion of judgments. In H. Guetzkow (Ed.), Groups, leadership, and men (pp. 177-190). Pittsburgh, PA: Carnegie Press.

Barry, C. L., Webster, D. W., Stone, E., Crifasi, C. K., Vernick, J. S., & McGinty, E. E. (2018). Public Support for Gun Violence Prevention Policies Among Gun Owners and Non–Gun Owners in 2017. *American journal of public health*, *108*(7), 878-881.

CDC "Fatal Injury Reports," Injury Prevention & Control: Data & Statistics (WISQARS), https://www.cdc.gov/injury/wisqars/fatal.html

Cohn, N. & Quealy, K. 2017. "Nothing Divides Voters Like Owning a Gun." The New York Times, 2017, October, 5. edition. https://www.nytimes.com/interactive/2017/10/05/upshot/gun-ownership-partisan divide.html.

De Vreese, C. H. (2004). The effects of frames in political television news on issue interpretation and frame salience. *Journalism & Mass Communication Quarterly*, *81*(1), 36-52.

Dixon, G. N., & Clarke, C. E. (2013). Heightening uncertainty around certain science: Media coverage, false balance, and the autism-vaccine controversy. *Science Communication*, *35*(3), 358-382.

Dixon, G. N., McKeever, B. W., Holton, A. E., Clarke, C., & Eosco, G. (2015). The power of a picture: Overcoming scientific misinformation by communicating weight-of-evidence information with visual exemplars. *Journal of Communication*, *65*(4), 639-659.

Drake, B. (2013). Gun rights proponents have been more politically active than gun control supporters. Pew Research. Retrieved from: https://www.pewresearch.org/fact tank/2013/03/29/gun-rights-proponents-have-been-more-politically-active-than-gun-control supporters/

Haines, M., & Spear, S. F. (1996). Changing the perception of the norm: A strategy to decrease binge drinking among college students. *Journal of American College Health, 45*(3), 134
-140.

Harvey, J. B. (1974). The Abilene paradox: The management of agreement. *Organizational Dynamics*.

Igielnik, R. & Brown, A. (2017). Key takeaways on Americans' views of guns and gun ownership. Pew Research Center. Retrieved from: http://www.pewresearch.org/fact tank/2017/06/22/key-takeaways-on-americans-views-of-guns-and-gun-ownership

Geiger, N., & Swim, J. K. (2016). Climate of silence: Pluralistic ignorance as a barrier to climate change discussion. *Journal of Environmental Psychology*, *47*, 79-90.Igielnik & Lamp

Leviston, Z., Walker, I., & Morwinski, S. (2013). Your opinion on climate change might not be as common as you think. *Nature Climate Change*, *3*(4), 334.

Lewandowsky, S., Gilles, G., & Vaughan, S. (2013). The pivotal role of perceived scientific consensus in acceptance of science. *Nature Climate Change, 3*, 399-404. doi:10.1038/10.1038/NCLIMATE1720.
© 2020 Dixon G et al. *JAMA Network Open.*

Miller, D. T., & McFarland, C. (1991). When social comparison goes awry: The case of pluralistic ignorance.

Parker, K., Horowitz, J., Igielnik, R., Oliphant, B., & Brown, A. (2017a). America's complex relationship with guns. Chapter 5. Views on guns. Retrieved from: http://www.pewsocialtrends.org/2017/06/22/views-on-gun-policy/

Parker, K., Horowitz, J., Igielnik, R., Oliphant, B., & Brown, A. (2017b). America's complex relationship with guns. An in-depth look at the attitudes and experiences of U.S. adults. Views on guns. Retrieved from:http://www.pewsocialtrends.org/2017/06/22/americas complex-relationship-with-guns/

Petersen, M. B., Skov, M., Serritzlew, S., & Ramsøy, T. (2013). Motivated reasoning and political parties: Evidence for increased processing in the face of party cues. *Political Behavior*, *35*(4), 831-854.

Prentice, D. A., & Miller, D. T. (1993). Pluralistic ignorance and alcohol use on campus: some consequences of misperceiving the social norm. *Journal of personality and social psychology*, *64*(2), 243.

Quinnipiac University Poll (2018). U.S. Support For Gun Control Tops 2-1, Highest Ever, Quinnipiac University National Poll Finds; Let Dreamers Stay, 80 Percent Of Voters Say. Retrieved from: https://poll.qu.edu/national/release-detail?ReleaseID=2521

Rios, K., & Chen, Z. (2014). Experimental evidence for minorities' hesitancy in reporting their opinions: The roles of optimal distinctiveness needs and normative influence. *Personality and Social Psychology Bulletin*, *40*(7), 872-883.

Joslyn, M. R., Haider-Markel, D. P., Baggs, M., & Bilbo, A. (2017). Emerging political identities? Gun ownership and voting in presidential elections. *Social Science Quarterly*, *98*(2), 382-396.

Schroeder, C. M., & Prentice, D. A. (1998). Exposing Pluralistic Ignorance to Reduce Alcohol Use Among College Students 1. *Journal of Applied Social Psychology*, *28*(23), 2150-2180.

Taylor, D. G. (1982). Pluralistic ignorance and the spiral of silence: A formal analysis. *Public Opinion Quarterly*, *46*(3), 311-335.

van der Linden, S. L., Leiserowitz, A. A., Feinberg, G. D., & Maibach, E. W. (2015). The scientific consensus on climate change as a gateway belief: Experimental evidence. *PloS one*, *10*(2), e0118489.

Williams, K. D. (2007). Ostracism. Annual Review of Psychology, 58(1), 425-452. doi:10.1146/annurev.psych.58.110405.085641

© 2020 Dixon G et al. *JAMA Network Open.*
